# GBP5 Repression Suppresses the Metastatic Potential and PD-L1 Expression in Triple-Negative Breast Cancer

**DOI:** 10.3390/biomedicines9040371

**Published:** 2021-04-01

**Authors:** Shun-Wen Cheng, Po-Chih Chen, Min-Hsuan Lin, Tzong-Rong Ger, Hui-Wen Chiu, Yuan-Feng Lin

**Affiliations:** 1 Department of Biomedical Engineering, Chung Yuan Christian University, Taoyuan City 32023, Taiwan; g9975606@cycu.edu.tw (S.-W.C.); sunbow@cycu.org.tw (T.-R.G.); 2 Neurology Department, Shuang-Ho Hospital, Taipei Medical University, New Taipei City 23561, Taiwan; d620100001@tmu.edu.tw; 3 Taipei Neuroscience Institute, Taipei Medical University, New Taipei City 23561, Taiwan; 4 Department of Neurology, School of Medicine, College of Medicine, Taipei Medical University, Taipei 11031, Taiwan; 5 Graduate Institute of Clinical Medicine, College of Medicine, Taipei Medical University, Taipei 11031, Taiwan; daisisky@yahoo.com.tw; 6 Department of Medical Research, Shuang Ho Hospital, Taipei Medical University, New Taipei City 23561, Taiwan; 7 TMU Research Center of Urology and Kidney, Taipei Medical University, Taipei 11031, Taiwan; 8 Cell Physiology and Molecular Image Research Center, Wan Fang Hospital, Taipei Medical University, Taipei 11696, Taiwan

**Keywords:** triple-negative breast cancer, metastasis, GBP5, IFN-γ, NF-κB, PD-L1

## Abstract

Triple-negative breast cancer (TNBC) is the most aggressive breast cancer subtype because of its high metastatic potential. Immune evasion due to aberrant expression of programmed cell death ligand 1 (PD-L1) has also been reported recently in metastatic TNBC. However, the mechanism underlying metastatic progression and PD-L1 upregulation in TNBC is still largely unknown. Here, we found that guanylate binding protein 5 (GBP5) is expressed in higher levels in TNBC tissues than in non-TNBC and normal mammary tissues and serves as a poorer prognostic marker in breast cancer patients. Transwell cultivation indicated that GBP5 expression is causally related to cellular migration ability in the detected TNBC cell lines. Moreover, the computational simulation of the gene set enrichment analysis (GSEA) program against the GBP5 signature generated from its coexpression with other somatic genes in TNBC revealed that GBP5 upregulation may be associated with the activation of interferon gamma (IFN-γ)-responsive and NF-κB-related signaling cascades. In addition, we found that the coexpression of GBP5 with PD-L1 was significantly positive correlation in TNBC tissues. Robustly, our data showed that GBP5 knockdown in TNBC cells harboring a higher GBP5 level dramatically suppresses the number of migrated cells, the activity of IFN-γ/STAT1 and TNF-α/NF-κB signaling axes, and the expression of PD-L1. Importantly, the signature combining a higher GBP5 and PD-L1 level predicted the shortest time interval of brain metastasis in breast cancer patients. These findings not only uncover the oncogenic function of GBP5 but also provide a new strategy to combat metastatic/immunosuppressive TNBC by targeting GBP5 activity.

## 1. Introduction

Triple-negative breast cancers (TNBCs) are immunohistochemically defined as a subset of breast cancers that are negative for human epidermal growth factor receptor-2 (HER2), estrogen receptor (ER) and progesterone receptor (PR) [1] and account for 15–20% of breast cancers [2]. TNBC is the most aggressive subtype in breast cancer with a highly metastatic capability and lack of specific targets and targeted therapeutics [3]. Although several biomarkers have been reported to predict brain, lung and bone metastasis of TNBC [4], distant metastasis of TNBC still severely risks the life of patients. On the other hand, immune evasion of TNBC via an increased level of programmed death-ligand 1 (PD-L1), which is an immune checkpoint inhibitor that interacts with PD-1 on the cell surface of T cells to suppress immune surveillance, has been reported as a route for the distant metastasis of TNBC [5,6]. Therefore, PD-L1 has been considered as an unavoidable biomarker in advanced TNBC [7]. However, the molecular mechanism by which metastatic TNBC upregulates the expression of PD-L1 is still largely unknown.

Guanylate binding protein 5 (GBP5) belongs to the family of interferon-gamma (IFN-γ)-inducible large GTPases and is responsible for many cellular functions, including inflammasome activation [8] and innate immunity against a wide variety of microbial pathogens [9,10,11,12]. The human GBP family consists of seven different members (GBP1-7) [13]. In addition to their functions in immune responses, it has been recently reported that GBP1 upregulation refers to a poor prognosis and probably regulates erlotinib resistance via phosphoglycerate kinase 1-mediated epithelial-mesenchymal transition (EMT) in lung adenocarcinoma [14]. Moreover, GBP1 knockout by the CRISPR/Cas9 system appeared to dramatically suppress the metastatic potential of prostate cancer cells [15]. In ER-negative breast cancer patients who developed brain metastasis, the GBP1 gene appeared to be upregulated by the stimulation of T lymphocytes, which promoted the ability of breast cancer cells to cross the blood–brain barrier [16]. GBP1 has also been proposed as a new potential therapeutic target for treating TNBC with enhanced EGFR expression [17]. GBP2 appeared to correlate with better prognosis in breast cancer and indicate an efficient T cell response [18]. GBP2 promoter methylation was found in TNBC and associated with advanced stages of breast cancer [19]. Except for GBP1 and GBP2, the role of other GBPs, including GBP5, in the malignant evolution of cancers, particularly TNBC, remains unclear.

Previous studies have demonstrated that GBP5 associated with good prognosis and correlated with immune infiltrations in PD-1 and PD-L1 high-expressing basal-like breast tumors [20]. Therefore, this study attempted to explore the functional roles of GBP5 in modulating the metastatic potential and PD-L1 expression in TNBC. Our results showed that GBP5 expression in TNBC is predominantly higher than that in non-TNBC and normal mammary tissues and is a poor prognostic marker in breast cancer patients. Moreover, GBP5 knockdown dramatically suppressed the cellular migration ability, PD-L1 expression, and activities of IFN-γ-responsive and NF-κB-regulated pathways in TNBC cells. Importantly, the signature of combining higher GBP5 and PD-L1 predicted a shorter time period for brain metastasis in breast cancer patients. These findings indicate a new strategy against metastatic TNBC by targeting GBP5 activity combined with immune checkpoint blockade therapy.

## 2. Materials and Methods

### 2.1. Clinicopathologic Results and Molecular Data for Breast Cancer Samples

The raw data of breast cancer patients and gene expression profiles of normal mammary and breast cancer tissues were obtained from the Gene Expression Omnibus (GEO) datasets GSE4922, GSE9195, GSE1379 and GSE12276 and The Cancer Genome Atlas (TCGA) database TCGA and GEO data were downloaded from the UCSC Xena website (UCSC Xena. Available online: http://xena.ucsc.edu/welcome-to-ucsc-xena/, accessed on 1 February 2021) and NCBI website, respectively. Microarray results of GSE4922, GSE9195, GSE1379 and GSE12276 datasets were further normalized by the median of mRNA expression levels from all samples and presented as log_2_ values. The gene lists of detected gene sets were downloaded from the Molecular Signature Database (https://www.gsea-msigdb.org/gsea/msigdb, accessed on 1 February 2021).

### 2.2. Cell Culture

TNBC cell lines HCC1806, HCC1937, MDA-MB-231 and Hs578T and embryonic kidney cell line 293T were obtained from American Type Culture Collection (ATCC). HCC1806 and HCC1937 cells were cultured in RPMI-1640 medium with 10% fetal bovine serum (FBS) at 37 °C with 5% CO_2_. Hs578T and 293T cells were cultivated in DMEM with 10% FBS. MDA-MB-231 cells were cultured in Leibovitz’s (L-15) medium with 10% FBS at 37 °C with 5% CO_2_. All media and supplements, e.g., FBS, were purchased from Gibco Life Technologies (Thermo Fisher Scientific Inc., Waltham, MA, USA). All cell lines used in this study were routinely authenticated on the basis of short tandem repeat (STR) analysis, mycoplasma detection and morphologic/growth characteristics.

### 2.3. Reverse Transcription PCR (RT-PCR) and Westen Blot Analyses

For RT-PCR experiments, TRIzol extraction kit purchased from Invitrogen (Thermo Fisher Scientific Inc., Waltham, MA, USA) was used to extract total RNA. RT-PCR experiments were performed by incubating total RNA (5 μg) with M-MLV reverse transcriptase (Invitrogen) and then amplifying cDNA products with Taq-polymerase (Protech) using paired primers (for GBP5, forward-GCCATTACGCAACCTGTAGTTGTG and reverse-CATTGTGCAGTAGGTCGATAGCAC; for PD-L1, forward-GCTGCACTTCAGATCACAGATGTG and reverse-GTGTTGATTCTCAGTGTGCTGGTC; for GAPDH, forward-AGGTCGGAGTCAACGGATTTG and reverse-GTGATGGCATGGACTGTGGTC).

For Western blot analyses, whole cell lysates obtained from designated experiments and TD-PM10315 TOOLS Pre-Stained Protein Marker (10–315 kDa) (BIOTOOLS Co., Ltd., Taipei, Taiwan) were separated by SDS-PAGE prior to transfer to PVDF membranes. Before the incubation with primary antibodies against GBP5 (GeneTex, Hsin-Chu, Taiwan, GTX118635, 1:1000) and GAPDH (AbFrontier, Seoul, Korea, #LF-PA0212, 1:5000) overnight at 4 °C, the membranes were immersed in the blocking buffer (5% nonfat milk in TBS containing 0.1% Tween-20) for 2 h at room temperature with a gentle agitation. After several washes, the membranes were incubated with peroxidase-labeled species-specific secondary antibodies for 1 h at room temperature. Immunoreactive bands were detected by an enhanced chemiluminescence system (Amersham Bioscience, Tokyo, Japan). Raw data of Western blot is shown in Figure S1.

### 2.4. Cellular Migration Assay

Cellular migration ability was determined by the trans-well cultivation using Boyden chambers (Neuro Probe, Inc., Gaithersburg, MD, USA). A polycarbonate membrane (8 μm pore size, 25 mm × 80 mm, Neuro Probe, Inc., USA) was precoated with 10 μg of human fibronectin (Sigma, MO, USA) on the side immersed at the lower chamber fulfilled with the conditioned medium (10% FBS). Cells (1.5 × 10^4^) were seeded in the top chamber containing 50 μL of starvation medium (0.1% FBS). For TNF-α effects, cells were preincubated with TNF-α (R&D Systems) at 10 ng/mL for 24 h prior to the trans-well cultivation. After the incubation for 4 h, the remaining cells on the top side of the membrane were removed prior to fixing the migrated cells on the bottom side of the membrane with 100% methanol followed by staining with 10% Giemsa’s solution (Merck, Germany) for 1 h. The migrated cells were finally counted under a microscope. All experiments were performed in triplicates and repeated three times.

### 2.5. Gene Knockdown and Reconstitution Experiments

Non-silencing (NS) control and GBP5 shRNA vectors containing a puromycin-resistant gene were obtained from the National RNAi Core Facility Platform in Taiwan. The package of lentiviral particles was performed by cotransfecting the NS control or GBP5 shRNA vector with the pMDG and pΔ8.91 constructs into 293T cells using a calcium phosphate transfection kit (Invitrogen). The media were harvested as viral stocks after the transfection for 48–72 h. Prior to the infection overnight with the generated lentiviral particles at a multiplicity of infection (MOI) of 2–10, cells at 50% confluence were preincubated with fresh media containing 5 μg/mL polybrene (Santa Cruz, Dallas, TX, USA) for 1 h. Cells stably expressing NS or GBP5 shRNA were selected after the cultivation in the conditioned media with puromycin at 10 μg/mL for 24 h. For GBP5 reconstitution, the GBP5-silenced MDA-MB-231 and Hs578T cells cultivated in the 10-cm plates were transfected with human GBP5 cDNA ORF clone (SinoBiological Inc., Wayne, PA, USA) at 1 and 3 μg using lipofectamine transfection procedure in accordance of the manufactural guideline (Invitrogen) for 24 h. The gene knockdown and restoration efficiency was validated by RT-PCR and Western blotting experiments.

### 2.6. Luciferase Reporter Assay

Luciferase reporter vectors harboring an IFN-gamma activation site (GAS) or NF-κB response element were obtained from Promega. Prior to the measurement of luciferase activity by a Dual-Glo^®^ Luciferase Assay System (Promega, Madison, WI, USA ), cell variants cultivated in 6-well plates were cotransfected with the tested luciferase reporter and Renilla luciferase-based control vectors for 24 h. After the transfection, whole cell lysates were obtained and then subjected to luciferase activity assay according to the manufacturer’s procedure. Finally, the luminescent intensity of firefly luciferase obtained from the detected cell variants was normalized to that of Renilla luciferase.

### 2.7. Statistical Analysis

All statistical analyses were analyzed by using SPSS 17.0 software (Informer Technologies, Roseau, Dominica). Nonparametric Spearman’s correlation test was performed to analyze the association among mRNA levels of GBP5, PD-L1 and IFN-γ gene set and immune checkpoint gene set in the detected samples. Kaplan–Meier analysis and log-rank tests were used to assess survival probabilities. One-way ANOVA with Tukey’s test, Student’s *t*-test and paired *t*-test and were used to analyze the statistical significance of the detected gene expression in clinical samples. The nonparametric Friedman test was used to determine the nonparametric data. In the all tests, *p* values < 0.05 were thought to be statistically significant.

## 3. Results

### 3.1. GBP5 Upregulation Correlates with Triple-Negative Characteristics and Poorer Prognosis in Breast Cancer

**Figure 1:**
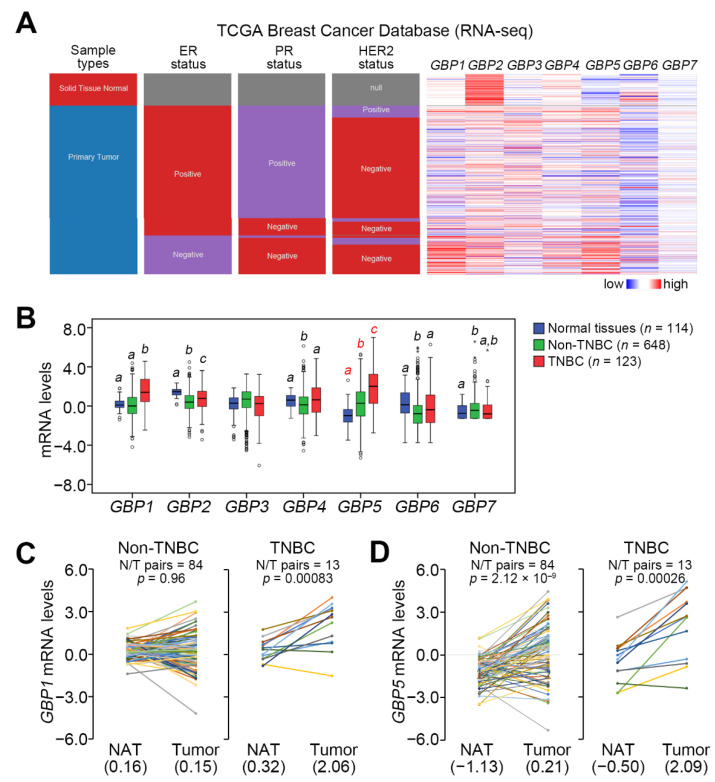
GBP5 (guanylate binding protein 5) is predominantly upregulated in TNBC (triple-negative breast cancers) compared to normal mammary and non-TNBC tissues. (**A**) Heatmap for the transcriptional profiling of genes encoding GBP1, GBP2, GBP3, GBP4, GBP5, GBP6 and GBP7 using the TCGA breast cancer database. (**B**) Boxplot of the mRNA levels of GBP1, GBP2, GBP3, GBP4, GBP5, GBP6 and GBP7 in normal tissues and primary tumor-derived non-TNBC and TNBC patients from the TCGA breast cancer database. Statistical significance was estimated by one-way ANOVA using Tukey’s post hoc test. Different letters indicate statistical significance at *p* < 0.01. (**C**,**D**) The mRNA levels of GBP1 (**C**) and GBP5 (**D**) in the paired normal adjacent tissues (NATs) and primary tumors, as shown in each colored lines, derived from TCGA non-TNBC and TNBC patients. The inserted values represent the median of GBP1 and GBP5 mRNA levels in NATs and primary tumors. The N/T pairs indicate NAT/primary tumor pairs. The statistical significance was evaluated by paired *t*-tests.

**Figure 2:**
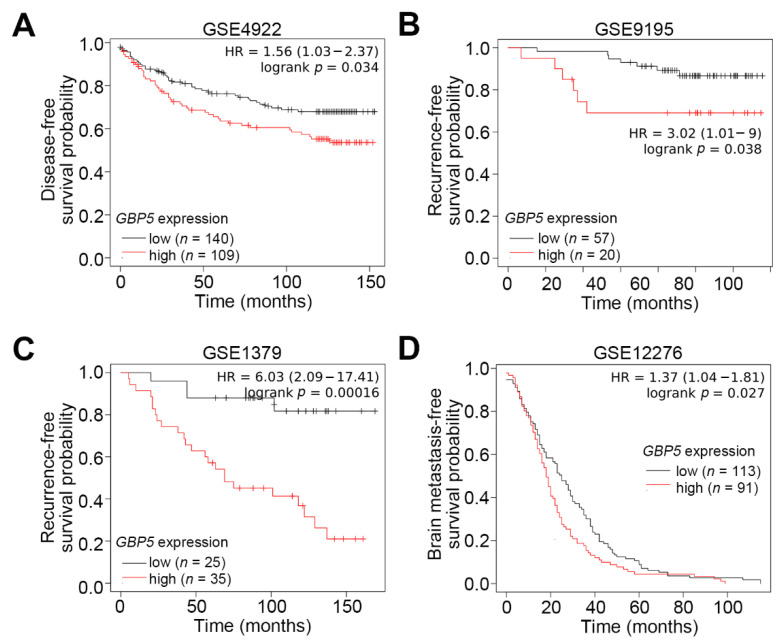
GBP5 is a poor prognostic marker in breast cancer patients. (**A**–**D**) Kaplan–Meier analyses for GBP5 transcripts using disease-free (**A**), recurrence-free (**B**,**C**) and brain metastasis-free (**D**) survival conditions under minimized log-rank *p* values against breast cancer patients derived from the GSE4922 (**A**), GSE9195 (**B**), GSE1379 (**C**) and GSE12276 (**D**) datasets.

We first dissected the expression of GBP members in the TCGA breast cancer database. Transcriptional profiling showed that the expression of GBP1 and GBP5, but not other GBPs, in TNBC tissues was significantly (*p* < 0.05) higher than that in non-TNBC and normal mammary tissues (Figure 1A,B). Moreover, the upregulation of GBP5 in primary tumors compared to normal adjacent tissues derived from non-TNBC and TNBC patients was more predominant than GBP1 (Figure 1C,D). Kaplan–Meier analyses revealed that GBP5 upregulation shows a poor disease-free survival rate in the GSE4922 breast cancer cohort, unfavorable recurrence-free survival probabilities in the GSE9195 and GSE1379 breast cancer cohorts and a shorter time period of brain metastasis in the GSE12276 breast cancer cohort (Figure 2A–D). These findings suggest that GBP5 upregulation may correlate with the malignant evolution of TNBC.

### 3.2. GBP5 Expression Is Causally Associated with Cellular Migration Ability in TNBC Cells

**Figure 3:**
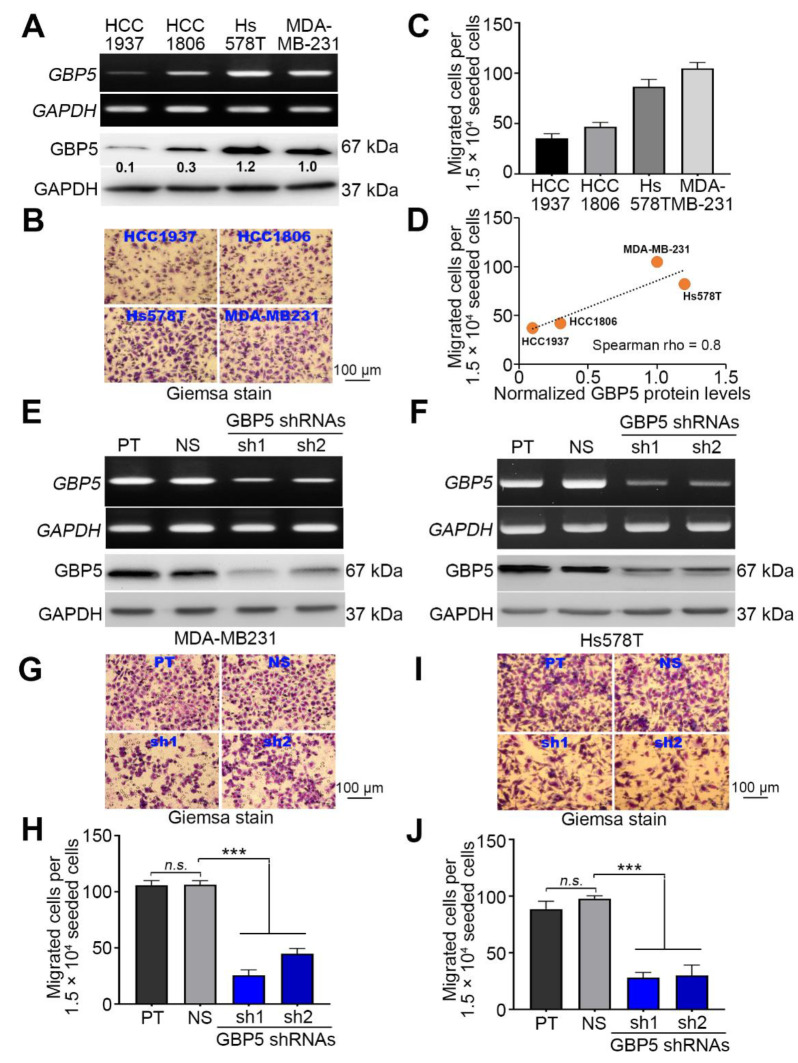
GBP5 knockdown suppresses the migration ability of TNBC cells. (**A**) The mRNA and protein levels of GAPDH and GBP5 detected by RT-PCR (upper panel) and Western blot (lower panel) analyses, respectively, in a panel of TNBC cell lines HCC1937, HCC1806, Hs578T and MDA-MB-231. (**B**,**C**) Geimsa staining (**B**) and the histogram (**C**) for the migrated cells of tested TNBC cell lines in Transwell cultivation for 4 h. (**D**) Dot plot for the correlation between normalized GBP5 protein levels, the inserts as shown Figure 3A, and migrated cell number in the tested TNBC cell line. The dashed line represents the regression line. (**E**,**F**) The mRNA and protein levels of GBP5 and GAPDH detected by RT-PCR (upper panel) and Western blot (lower panel) analyses, respectively, in parental (PT) MDA-MB-231 (**E**)/Hs578T (**F**) cells and MDA-MB-231/Hs578T cells transfected with nonsilencing (NS) control shRNA or 2 independent GBP5 shRNAs. In (**A**,**E**,**F**), GAPDH was used as an internal control for experiments. (**G**,**J**) Geimsa staining (**G**) and (**I**) and the histogram (**H**) and (**J**) for the migrated cells of tested MDA-MB-231 (**G,H**) and Hs578T (**I,J**) cell variants in Transwell cultivation for 4 h. In (**C**,**H**,**J**), the error bars denote the data from three independent experiments presented as the mean ± SEM. A nonparametric Friedman test was used to analyze the statistical significance. The symbols “***” and “n.s.” denote *p* < 0.001 and not significant, respectively.

Since TNBC is a highly metastatic subtype of breast cancer, we next determined the correlation between GBP5 expression and cellular migration ability in the TNBC cell lines HCC1937, HCC1806, Hs578T and MDA-MB-231. RT-PCR and Western blotting results demonstrated that GBP5 mRNA and protein expression in Hs578T and MDA-MB-231 cells was predominantly higher than that in HCC1937 and HCC1806 cells (Figure 3A). Accordingly, a cell migration assay revealed that the migration ability of Hs578T and MDA-MB-231 cells was greater than that of HCC1937 and HCC1806 cells (Figure 3B,C). The endogenous GBP5 protein levels and cellular migration abilities in the tested TNBC cell lines appeared to be positively correlated (Figure 3D). Moreover, the gene knockdown of GBP5 by its two independent shRNA clones dramatically repressed the endogenous mRNA level of GBP5 in MDA-MB-231 and Hs578T cells compared to the parental and nonsilencing control cells (Figure 3E,F). Similarly, GBP5 knockdown appeared to significantly (*p* < 0.001) suppress the migration ability of MDA-MB-231 and Hs578T cells compared to the parental and nonsilencing control cells (Figure 3G–J).

### 3.3. GBP5 Upregulation Probably Correlates with Elevated Activities of IFN-γ and NF-κB-Related Signaling Pathways in TNBC

**Figure 4:**
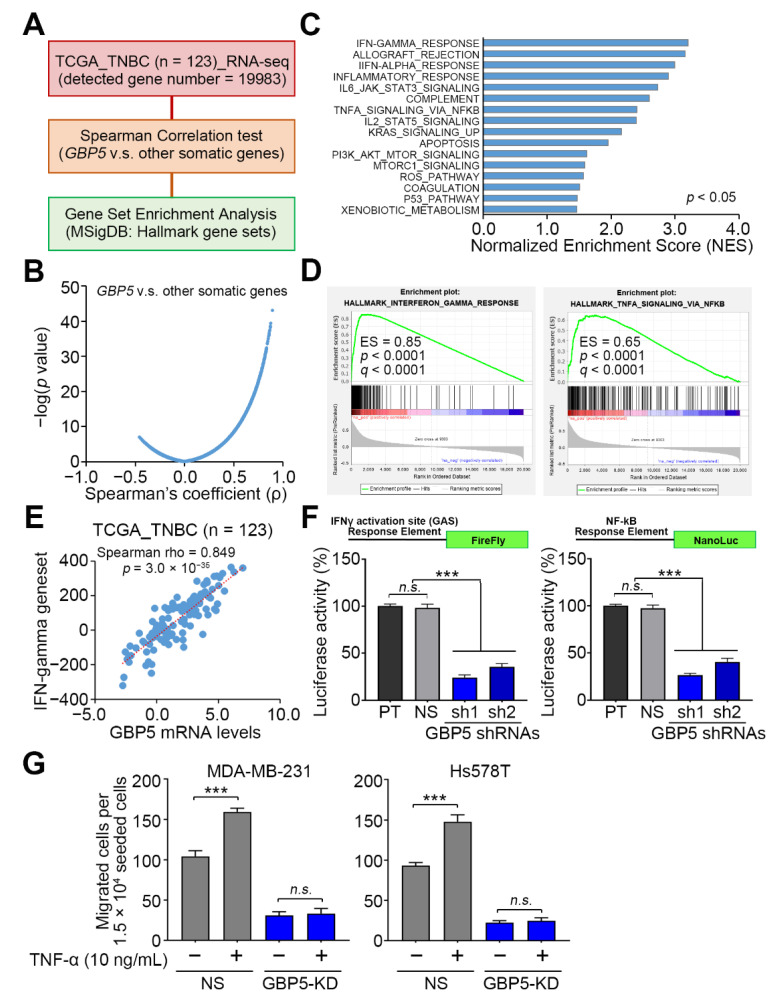
GBP5 knockdown downregulates the activity of IFN-*γ* and NF-*κB* in TNBC cells. (**A**) Flow chart of generating the GBP5 signature from TNBC samples from the TCGA database for GSEA simulation. (**B**) A volcano plot for the Spearman’s coefficient (*p*) values and—log (*p* values) derived from the Spearman correlation test against the coexpression of GBP5 with other somatic genes detected by RNA-sequencing tool in 123 TNBC samples deposited in the TCGA database. (**C**) A histogram for normalized enrichment scores derived from GSEA simulation using hallmark gene sets deposited in the molecular signature database (MSigDB) against the GBP5 signature. (**D**) The enrichment score (ES) derived from the correlation of the GBP5 signature with the IFN-*γ*-responsive (left) and TNF-*α*/NF-*κ*B signaling axis-related (right) gene signatures was plotted as the green curve. The parameters of the enrichment score, nominal *p* value and false discovery rate q value are shown as insets. (**E**) Scatchard plot for the expression of GBP5- and IFN-*γ*-responsive gene sets in TNBC samples from the TCGA database. The Spearman correlation test was used to evaluate the statistical significance. The dashed/red line represents the regression line. (**F**) Constructs of the firefly luciferase gene adjacent to the promoter harboring the IFN-*γ* activation site (GAS) response element (upper left) and NanoLuc luciferase gene adjacent to the promoter harboring the NF-*κ*B response element (upper right), and histograms of the firefly (lower left) and NanoLuc (lower right) luciferase activities detected in the indicated MDA-MB-231 cell variants. (**G**) The histograms for the migrated cells in the non-silencing control and GBP5-knockdown (GBP5-KD) MDA-MB-231 (left) and Hs578T (right) cells without or with TNF-α pretreatment at 10 ng/mL for 24 h. In (**F**,**G**), the error bars denote the data from three independent experiments presented as the mean ± SEM. A nonparametric Friedman test was used to analyze the statistical significance. The symbols “***” and “n.s.” represent *p* < 0.001 and not significant, respectively.

To understand the possible mechanism by which GBP5 upregulation enhances the metastatic potential of TNBC, we next performed a computational simulation by using the gene set enrichment analysis (GSEA) program. To obtain a GBP5-related signature, we first performed Spearman’s correlation tests against the coexpression of GBP5 with other somatic genes and determined the RNA-sequencing method in TNBC samples deposited in the TCGA database (Figure 4A). Then, the ranked Spearman’s coefficient ρ values were used as a GBP5-related signature for further GSEA simulation (Figure 4B). GSEA results revealed that the GBP5 signature highly correlates with the IFN-γ-responsive pathway in TNBC (Figure 4C–E), which is consistent with previous reports that GBP5 is an IFN-γ-inducible gene [10,21,22]. In addition, computational simulation indicated that the GBP5 signature is closely associated with the activation of inflammation-related pathways, such as the IL-6/JAK/STAT3 and TNF-α/NF-κB signaling cascades, in TNBC (Figure 4C,D), which is similar to the previous finding that GBP5 promotes inflammasome assembly in macrophages [8]. As a result, we next performed a luciferase-based reporter assays for determining the DNA-binding activities of IFN-γ-responsive STAT1 and TNF-α-activated NF-κB towards the IFN-*γ* activation site (GAS) response element adjacent to a firefly luciferase gene and the NF-*κ*B response element adjacent to a NanoLuc luciferase gene, respectively, in MDA-MB-231 cells. Luciferase reporter assays demonstrated that GBP5 knockdown via its two independent shRNA clones significantly (*p* < 0.001) suppressed the activity of the IFN-γ-responsive signaling axis, and the DNA-binding activity of NF-κB, in MDA-MB-231 cells (Figure 4F). Robustly, the pretreatment of TNF-α potentiated the cellular migration ability in the non-silencing control MDA-MB-231 and Hs578T cells, but not in the GBP5-knockdown MDA-MB-231 and Hs578T cells (Figure 4G). These findings may suggest that GBP5 governs the activity of TNF-α/NF-κB signaling axis in TNBC cells.

### 3.4. GBP5 Repression Reduces the Expression of PD-L1 in TNBC, and Its Upregulation Predicts a Shorter Time Interval for Brain Metastasis of Breast Cancer

**Figure 5:**
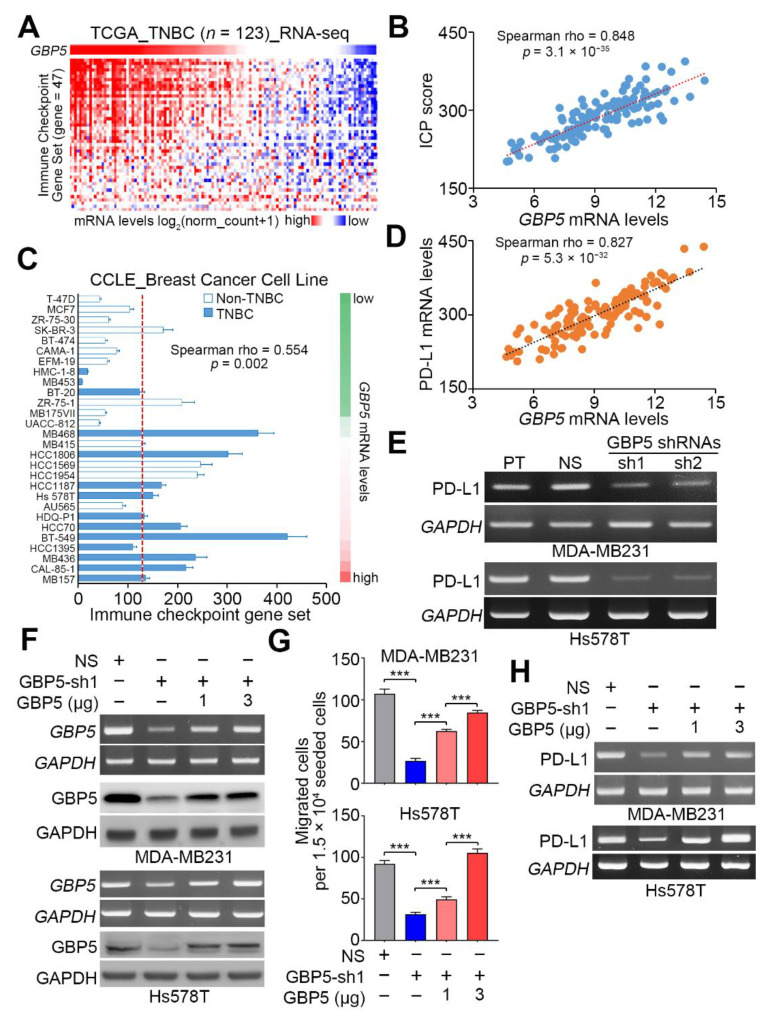
GBP5 knockdown reduces the expression of PD-L1 in TNBC cells. (**A**) Heatmap for the transcriptional profiling of GBP5 and the immune checkpoint (ICP) gene set detected by the RNA-sequencing (RNA-seq) tool in TNBC samples from the TCGA database. (**B**) Scatchard plot for the expression of the GBP5 and ICP gene sets in TNBC samples from the TCGA database. (**C**) Histogram for the expression of the GBP5 and ICP gene sets in the non-TNBC and TNBC cell lines deposited in the GSE36133 dataset. (**D**) Scatchard plot for the expression of GBP5 and PD-L1 in TNBC samples from the TCGA database. In B, C and D, the Spearman correlation test was used to evaluate the statistical significance. In B and D, the dashed lines represent regression line. (**E**) The mRNA levels of PD-L1 and GAPDH detected by RT-PCR in parental (PT) MDA-MB-231 (upper panel)/Hs578T (lower panel) cells and MDA-MB-231/Hs578T cells transfected with nonsilencing (NS) control shRNA or 2 independent GBP5 shRNAs. (**F**) The mRNA and protein levels of GBP5 and GAPDH determined by RT-PCR and Western blotting, respectively, in the NS control and GBP5-sh1-silenced MDA-MB-231 (upper panel) and Hs578T (lower panel) cells without or with the restoration of exogenous GBP5 DNA at 1 and 3 μg. (**G**) The histograms for the migrated cells in the MDA-MB-231 (upper panel) and Hs578T (lower panel) cell variants as shown in F. The error bars denote the data from three independent experiments presented as the mean ± SEM. A nonparametric Friedman test was used to analyze the statistical significance. The symbol “***” represent *p* < 0.001. (**H**) The mRNA levels of PD-L1 and GAPDH detected by RT-PCR in the MDA-MB-231 (upper panel) and Hs578T (lower panel) cell variants as shown in (**F**). In (**E**,**F**,**H**), GAPDH was used as an internal control of experiments.

**Figure 6:**
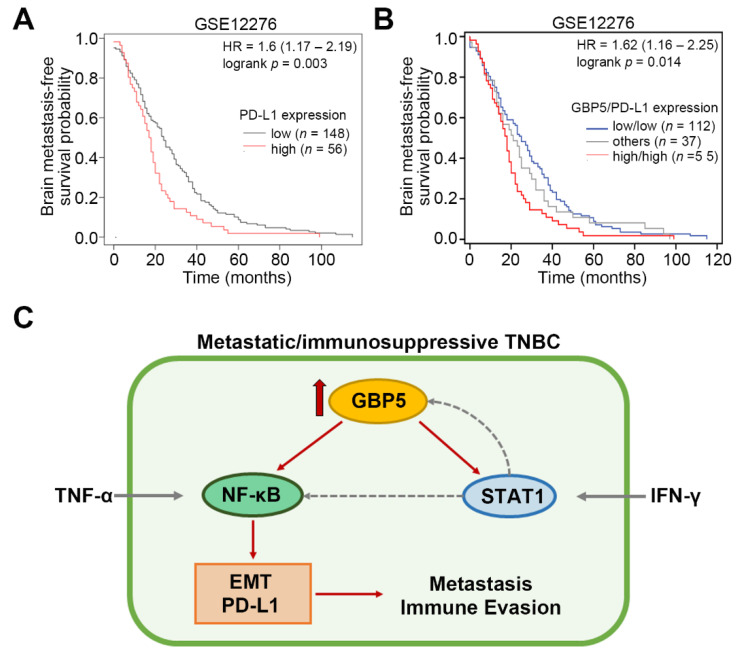
The signature combining higher levels of GBP5 and PD-L1 transcripts correlates with the shortest time interval for brain metastasis in breast cancer patients. (**A** and **B**) Kaplan–Meier analyses for PD-L1 transcripts without (**A**) or with (**B**) the combination of GBP5 expression using disease-free brain metastasis-free survival conditions under a minimized log-rank *p* value against breast cancer patients derived from GSE12276 datasets. (**C**) A possible mechanism for GBP5-promoted metastasis and immunosuppression in TNBC.

Since GBP5 expression is likely associated with immune modulation in TNBC, we next examined if GBP5 is capable of regulating the activity of immune checkpoints. We first dissected the transcriptional profiling of GBP5 and the immune checkpoint (ICP) gene set [23] in TNBC samples from the TCGA database (Figure 5A). The data showed that the correlation between GBP5 transcripts and the mRNA levels of the ICP gene set was strongly positive in the detected TNBC samples (Figure 5B). Compared to non-TNBC cell lines, the mRNA levels of the ICP gene set were relatively higher in TNBC cell lines and positively correlated with GBP5 expression (Figure 5C). Furthermore, we found that the coexpression of GBP5 and PD-L1 was highly positive with great statistical significance (*p* = 5.3 × 10^−32^) in the detected TNBC samples (Figure 5D). Robustly, GBP5 knockdown repressed the mRNA levels of PD-L1 in Hs578T and MDA-MB-231 cells (Figure 5E,F). To ascertain if GBP5 acts as a critical molecule in promoting the metastatic progression and PD-L1 expression in TNBC, we next performed the reconstitution of GBP5 expression in the GBP5-silenced MDA-MB-231 and Hs578T cells. The data showed that the restoration of GBP5 levels by transfecting the GBP5-silenced MDA-MB-231 and Hs578T cell with exogenous GBP5 DNA at 1 and 3 μg predominantly elevates the intracellular mRNA and protein levels of GBP5 (Figure 5F) and enhances the cellular migration ability (Figure 5G) and PD-L1 expression (Figure 5H) in a concentration-dependent manner. Moreover, Kaplan–Meier analyses showed that a higher mRNA level of PD-L1 indicated a poor brain metastasis-free probability in the GSE11276 breast cancer cohort (Figure 6A). Importantly, the signature combining high-level GBP5 and PD-L1 transcripts predicted the shortest time interval for brain metastasis in breast cancer patients from the GSE11276 dataset (Figure 6B).

Collectively, we proposed that GBP5 upregulation probably enhances the activity of IFN-γ/STAT1 and TNF-α/NF-κB signaling cascades in TNBC cells. The activated IFN-γ/STAT1 signaling axis may construct a positive feedback loop to reinforce GBP5 function because GBP5 is an IFN-γ-responsive effector [21]. Moreover, the activated IFN-γ/STAT1 signaling axis may also potentiate NF-κB activity because the coordination of IFN-γ with TNF-α in activating NF-κB has also been reported previously [24]. Since NF-κB has been shown to regulate EMT and PD-L1 expression in lung cancer [25], we thus thought that enforcedly activated NF-κB further triggers the progression of EMT and the expression of PD-L1, thereby ultimately fostering the metastasis and immunosuppression of TNBC (Figure 6C).

## 4. Discussion

Previous reports have shown that GBP1 upregulation predicts a poorer prognosis in different types of cancer, including TNBC, whereas a higher level of GBP2 correlates with a favorable outcome in breast cancer. Here, we further show that GBP5 could serve as a poor prognostic biomarker in TNBC. Moreover, GBP5 upregulation may be associated with cancer progression, e.g., metastasis, in TNBC. Notably, a higher level of GBP5 appeared to correlate with a shorter time period for brain metastasis in breast cancer patients. Although the incidence of brain metastasis in the TNBC subtype is approximately 46% [26], the prognostic significance of GBP5 to predict the brain metastasis of TNBC still needs to be further validated.

The biological activity of IFN-γ is associated with cytostatic/cytotoxic and antitumor mechanisms during the cell-mediated adaptive immune response. However, tumor specificity, signaling intensity and microenvironmental factors were recently found to confer protumorigenic activity of IFN-γ [27]. Here, we found that the GBP5 signature derived from its coexpression with other somatic genes in TNBC samples from the TCGA database was highly associated with the activation of the IFN-γ-responsive pathway. Moreover, GBP5 knockdown appeared to suppress the activity of the IFN-γ-dependent signaling axis, as judged by STAT1 binding to the gamma-activated sequence (GAS) within the promoter region of the luciferase gene and ultimately reduced the cellular migration ability of TNBC cells. Although the molecular mechanism by which GBP5 fosters STAT1 activity to promote the metastatic progression of TNBC needs to be further explored, this is the first study to document that the IFN-γ-responsive gene GBP5 is capable of reinforcing the IFN-γ-dependent signaling axis in TNBC.

It has been found that the induction of inflammation-related pathways promotes metastatic progression in breast cancer [28,29]. NF-κB is recognized as a key transcription factor in regulating inflammation-related gene expression [30], thereby enhancing the metastatic potential of TNBC [31,32,33]. Moreover, the cross-talk between NF-κB and the IL-6/STAT3 axis was also found to confer doxorubicin resistance in TNBC MDA-MB-231 cells [34]. Here, we show that GBP5 upregulation may be associated with the activation of the TNF-α/NF-κB and IL-6/STAT3 signaling pathways in TNBC. GBP5 knockdown appeared to suppress the activity of NF-κB in MDA-MB-231 cells. Although further investigations still need to validate the role of GBP5 in driving TNBC metastasis, the involvement of GBP5 in NLRP3-mediated inflammasome assembly [21] may indicate its pivotal role in governing inflammation-responsive pathways during the metastatic progression of TNBC.

PD-L1 expression was detected in approximately 20% of TNBCs and is thought to be a therapeutic target for treating TNBC patients [6]. A phase I study revealed that 24% of patients with metastatic TNBC in the trial of MPDL3280A, a monoclonal antibody against PD-L1, show complete or partial responses in the average follow-up of 40 weeks [5]. PD-L1 blockade was found to suppress the metastatic potential of MDA-MB-231 cells by inhibiting the activity of NF-κB [35]. Intriguingly, the activation of the TNF-α/NF-κB signaling axis has been shown to promote EMT progression and PD-L1 expression in lung cancer [25]. These findings suggest that PD-L1 probably constructs a positive feedback loop to reinforce the activity of the TNF-α/NF-κB signaling axis in metastatic TNBC. In this study, we found that GBP5 knockdown is capable of repressing PD-L1 levels and suppressing the activity of NF-κB in TNBC cells, suggesting a therapeutic value of targeting GBP5 in combating metastatic TNBC.

## 5. Conclusions

This study is the first to demonstrate the oncogenic role of GBP5 in promoting the metastatic progression of TNBC by activating pathways related to IFN-γ and inflammatory responses. Since IFN-γ and TNF-α have been shown to synergistically regulate NF-κB activity for the expression of IL-8 in gastric cancer cells [24], our findings provide a new strategy to combat metastatic TNBC by targeting GBP5 activity, which could downregulate NF-κB-mediated PD-L1 expression, thereby enhancing tumor immune surveillance. Moreover, our results also suggest that GBP5 upregulation may serve as an indicator for PD-L1/PD-1 immune checkpoint blockade therapy against TNBC.

## Data Availability

The data presented in this study are available in article.

## Supplementary Materials

The following are available online at https://www.mdpi.com/article/10.3390/biomedicines9040371/s1, Figure S1: uncut blots for Figure 3.
