# Supplementary figures and images for: GBP5 Repression Suppresses the Metastatic Potential and PD-L1 Expression in Triple-Negative Breast Cancer
Source: Biomedicines. 2021 Apr 1;9(4):371. doi: 10.3390/biomedicines9040371 (PMC8066311; doi:10.3390/biomedicines9040371)

**Figure 3A**

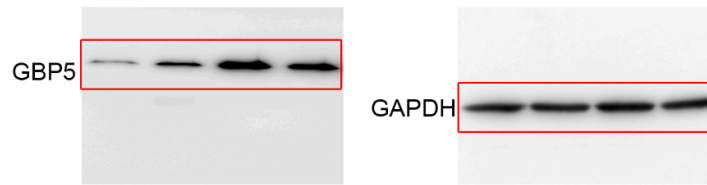

**Figure 3E**

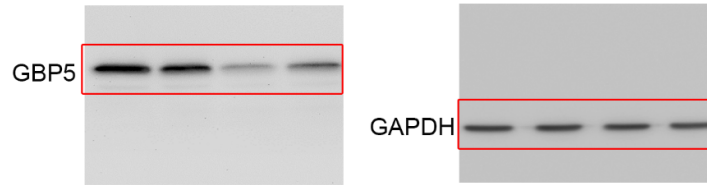

**Figure 3F**

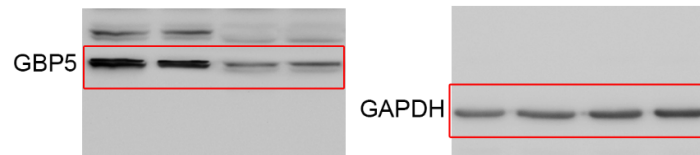

**Figure 5F**

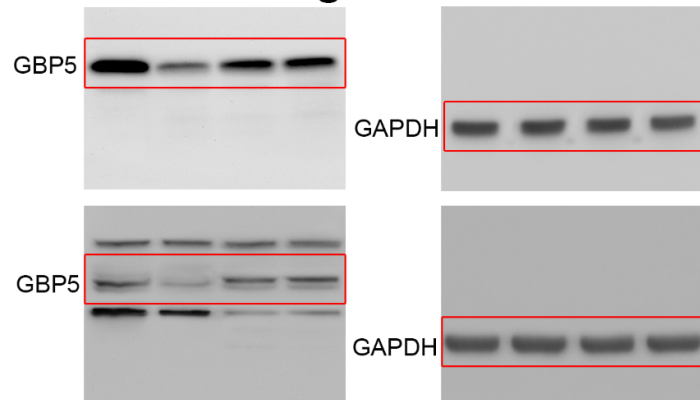

**Figure S1.** Raw data of Western blot. Raw data for Figures 3A,E,F and 5F.

Supplement: Supplementary file 1 [file biomedicines-09-00371-s001.pdf]
